# Energy Transfer Mechanism of Hard-Roof Hydraulic Fracturing in Goaf-Side Working Face Based on Microseismic-Driven Damage Model

**DOI:** 10.3390/s26113566

**Published:** 2026-06-03

**Authors:** Rupei Zhang, Siyuan Gong, Wu Cai, Hui Li, Yuanhang Qiu

**Affiliations:** 1 School of Mines, China University of Mining and Technology, Xuzhou 221116, China; 2 School of Computer Science and Technology/School of Artificial Intelligence, China University of Mining and Technology, Xuzhou 221116, China

**Keywords:** hydraulic fracturing, rockburst, microseismic monitoring, rock damage, finite-difference simulation

## Abstract

**Highlights:**

**What are the main findings?**

- A microseismic-energy–Benioff-strain–Weibull-damage-based characterization method for hydraulic fracturing is established, which can represent the heterogeneous weakening state of the target roof in a FLAC3D finite-difference model.
- Numerical simulations indicate that a high-damage weakening zone forms ahead of the goaf-side working face, reducing the strength and local bearing capacity of the fractured horizon, thereby altering the mining-induced load-transfer path.

**What are the implication of the main findings?**

- Hydraulic fracturing transforms continuous high stress and high energy zones into localized and dispersed distributions, weakening the high static-load and high-energy-storage state of the coal-rock mass ahead of the working face.
- The rockburst prevention mechanism of roof hydraulic fracturing relies on bearing-structure reconstruction and load-path adjustment under the control of the heterogeneous damage zone formed in the fractured horizon.

**Abstract:**

Directional long-borehole hydraulic fracturing is an important technique for controlling rockbursts induced by hard roofs. Its effectiveness depends primarily on whether fracturing-induced damage can modify the roof-bearing structure and thereby regulate stress concentration and elastic strain energy accumulation in the coal-rock mass ahead of the working face. However, existing numerical simulations commonly rely on predefined weakened zones or empirical parameter reduction, which makes it difficult to represent the spatial heterogeneity and mechanical evolution of rock damage during field hydraulic fracturing. Taking the 2803 goaf-side working face in Hetaoyu Coal Mine as the engineering background, this study proposes a microseismic-data-driven method for characterizing hydraulic fracturing-induced damage and incorporates it into a FLAC3D finite-difference model. The stress field, elastic strain energy field, and damage distribution ahead of the working face are compared under non-fractured and hydraulically fractured conditions. In the proposed method, the energy of fracturing-induced microseismic events is converted into the Benioff strain of numerical zones according to the attenuation law of microseismic wave propagation, and the corresponding rock damage variable is then calculated using a Weibull damage model. The fracturing-damaged rock mass is further represented by weakening the elastic modulus, cohesion, and friction angle, together with the stochastic generation of strongly damaged zones. The results show that, without hydraulic fracturing, the hard roof maintains a strong, continuous bearing capacity, resulting in a continuous lateral abutment stress concentration zone and a high elastic strain energy accumulation zone ahead of the working face and near the goaf-side boundary. After hydraulic fracturing, a patchy and locally connected high-damage weakening zone forms in the target roof strata. This damaged zone cuts the original continuous load-transfer structure through which the hard roof concentrates load toward the goaf side, reduces the extent of high-stress and high-energy zones in the coal seam, and induces an asymmetric adjustment of the dominant mining-induced energy release zone from the goaf side toward the solid-coal side. These simulation results agree well with the field observation that microseismic activity is mainly concentrated near the roadway on the solid-coal side. The study indicates that the rockburst-control mechanism of directional long-borehole hydraulic fracturing is not limited to simple overall stress dissipation. A key finding is that the fracturing-induced heterogeneous damage zone effectively interrupts the continuous load-transfer and energy-storage paths on the goaf side. This induces an asymmetric spatial redistribution of the mining-induced energy field from the goaf side toward the solid-coal side, thereby mitigating the high static-load and high-energy-storage state ahead of the working face.

## 1. Introduction

With the continuous increase in mining depth and mining intensity in China, rockbursts have become increasingly frequent and now represent one of the major hazards restricting safe coal extraction. The complexity of underground mining environments leads to diverse triggering factors for rockbursts. In China, a large proportion of rockburst-prone mines are affected by hard roofs. Hard roofs are characterized by high strength, large breaking intervals, wide influence ranges, intense ground pressure behavior, and complex mechanical responses, making them a major challenge in roof control. Rockburst manifestations induced by hard roofs are mainly associated with stress concentration caused by suspended roofs and dynamic disturbance generated by roof breakage, both of which can trigger coal-rock failure [1,2,3,4]. Moreover, hard roofs can accumulate a large amount of elastic strain energy and act as a seismic source layer. Once fractured, they may release substantial energy and generate mining-induced seismicity, thereby inducing dynamic coal-rock disasters in mining spaces [5]. Previous studies have shown that the continuous bearing capacity of hard roofs provides the structural basis for high elastic strain energy accumulation ahead of the working face. Therefore, pre-fracturing hard roofs and weakening their structural integrity are important measures for rockburst prevention. Against this background, hydraulic fracturing has been increasingly applied to the prevention and control of hard-roof-induced rockbursts because of its operational simplicity, broad adaptability, and relatively high safety [6].

The key function of hydraulic fracturing in rockburst control is to promote fracture propagation and structural weakening in hard roofs through high-pressure water injection, thereby modifying their intact bearing state and failure mode. On the one hand, hydraulic fractures reduce the integrity and strength of hard roofs, shorten the effective suspended roof length, and promote the transition of roof failure from large-scale integral breakage to segmented or progressive breakage. This process can reduce dynamic disturbance caused by sudden large-scale roof instability [7,8]. On the other hand, the fractured zone changes the continuity of mining-induced load transfer toward the coal seam and roadway surrounding rock, weakening the formation conditions of highly concentrated stress zones and reducing the capacity of the coal-rock mass to accumulate elastic strain energy [9]. Meanwhile, hydraulic fracturing can also promote early local energy release, transforming microseismic activity from a small number of high-energy events to a larger number of low-energy events, thereby reducing the likelihood of high-energy microseismic events or rockbursts [10,11]. Therefore, the rockburst-control effect of hydraulic fracturing does not rely solely on fracture creation. Rather, it is achieved through the combined regulation of roof weakening, load redistribution, and energy release.

With the continuous improvement of computational capacity, numerical modeling has become an important tool in geotechnical engineering analysis and design. Numerous studies have investigated hydraulic fracturing-based hard roof control using numerical simulation and have obtained useful results. Existing studies have mainly focused on hydraulic fracture propagation, roof weakening effects, mining-induced stress redistribution, and fracturing parameter optimization. These studies indicate that numerical models can effectively characterize the propagation morphology of hydraulic fractures in hard roofs and their interaction with bedding planes and weak discontinuities. They can also be used to analyze post-fracturing roof caving, goaf compaction, and changes in abutment pressure ahead of the working face. Zhang Hongwei et al. [12] established a coupled simulation method for “hydraulic fracturing + pressure-relief mining” using the discrete element method and analyzed the effects of fracturing fluid rate, bedding weakness, and fracture propagation on hard-roof pressure-relief mining. They reported that hydraulic fracturing promoted earlier and more complete caving of the hard roof and improved the suspended roof structure. Cao et al. [13] combined numerical simulation and field tests to investigate the influence of different fracturing parameters on hard-roof weakening and suggested that appropriate hydraulic fracturing parameters can effectively reduce hard-roof pressure and improve surrounding rock response in the stope. Yu et al. [14] investigated ground hydraulic fracturing technology and its engineering application for large-space hard roof control, showing that ground fracturing can effectively modify hard roof structures and improve roof control in large-space stopes. Wang et al. [15] analyzed the weakening mechanism and the fracability evaluation method of deep thick hard strata under fracturing control, providing a theoretical basis for rockburst prevention under deep high-stress conditions. Pan et al. [16] proposed the “artificial liberated layer” method for rockburst prevention and explained its mechanism in reducing burst risk by artificially weakening key roof structures and releasing or transferring high-level loads. These studies demonstrate the applicability of hydraulic fracturing in hard-roof-induced rockburst prevention from the perspectives of engineering practice, weakening mechanisms, and prevention methods, and they also provide an important basis for subsequent numerical modelling studies. Xia et al. [17] used CDEM numerical simulation to analyze the pressure-relief and rockburst-control effects of staged fracturing in ultra-long horizontal boreholes for weakening thick hard roofs. Zhuang et al. [18] established a UDEC numerical model to reproduce the evolution of mining-induced stress and high-energy microseismic events after hydraulic fracturing and clarified the mechanism by which hydraulic fracturing controls rockbursts. Their results showed that hydraulic fracturing significantly weakened the structural integrity of hard roofs, promoted timely roof caving, and reduced the additional load caused by suspended roof structures. After hydraulic fracturing, the total frequency and total released energy of microseismic events increased, whereas the average energy of individual events decreased significantly. Zou et al. [19] combined field monitoring and PFC numerical simulation to systematically analyze the evolution of overburden fracture characteristics and microseismic distribution before and after hydraulic fracturing, revealing the effect of hydraulic fracturing in controlling high-energy microseismic events. Their numerical results showed that hydraulic fracturing can effectively weaken the integrity of thick hard strata, induce their fracture, and thereby reduce or eliminate large-energy hazardous microseismic events.

However, for the rockburst-control mechanism of hydraulic fracturing under the complex stress environment of deep goaf-side working faces, several significant gaps remain. Previous numerical studies on fracturing-induced pressure relief have mostly treated a single working face as an isolated object, often simplified as a two-dimensional plane model, while neglecting the constraints imposed by the actual surrounding mining environment. Such idealized symmetric boundary conditions fundamentally obscure the asymmetric stress concentration characteristic of goaf-side conditions and make it difficult to examine how hydraulic fracturing intervenes in this asymmetric high-energy evolution process. In terms of modeling approaches, existing studies have mostly used discrete element methods to reproduce the microscopic morphology of fracture propagation. By contrast, the Lagrangian finite-difference method, which is better suited to three-dimensional macroscopic load redistribution and energy-flow analysis in continuous media, has been less frequently applied to such large-scale spatiotemporal evolution problems. In addition, conventional simulations often separate field monitoring from numerical calculation and rely on manually predefined homogeneous weakened zones. The lack of a damage model driven by real microseismic data seriously limits the quantitative understanding of mining-induced energy redistribution controlled by fracturing-induced damage zones. Therefore, taking a typical deep goaf-side working face and its adjacent goaf as a three-dimensional engineering background, this study directly maps field microseismic data collected during hydraulic fracturing into a FLAC3D finite-difference model based on continuum damage mechanics, thereby establishing a data-driven numerical analysis framework. The objective is to reveal the redistribution characteristics of subsequent mining-induced stress flow and elastic strain energy under the control of heterogeneous damage zones, and to clarify the rockburst-control mechanism by which mining-induced energy is spatially transferred toward the solid-coal side after hydraulic fracturing of a hard roof. The results are expected to provide a more rigorous theoretical basis and technical support for rockburst prevention in deep coal mines.

## 2. Engineering Background

### 2.1. Overview of the Working Face

The 2803 working face (LW2803) is located on the western side of the 2804 working face (LW2804) in Panel II of the southern wing of Hetaoyu Coal Mine. An 8 m wide coal pillar is retained between the two working faces. The strike length and dip length of the LW2803 are 2100 m and 240 m, respectively. The average coal seam thickness is 12.5 m, the average dip angle is 3°, and the burial depth ranges from 977.48 m to 1006.50 m.

**Figure 1:**
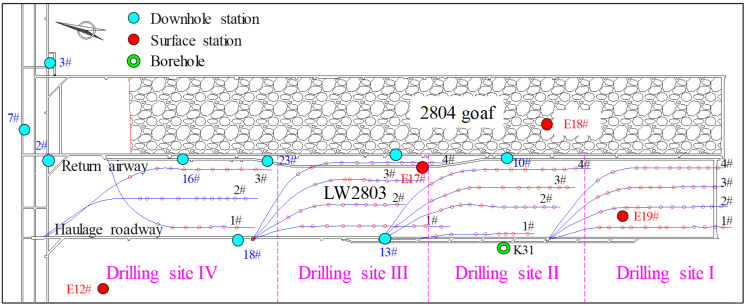
Layout of LW2803 and microseismic monitoring network.

Hetaoyu Coal Mine is equipped with an integrated underground-surface SOS microseismic monitoring system. This system consists of an underground SOS microseismic monitoring network and a surface monitoring network incorporating 4G communication, GPS timing, and three-component sensing technologies. The layouts of the underground and surface monitoring networks during a representative mining period of the LW2803 are shown in Figure 1. Owing to the three-dimensional enclosure provided by the integrated monitoring network, the accuracy and reliability of microseismic monitoring data in the nearly horizontal coal seam are effectively ensured [20].

**Figure 2:**
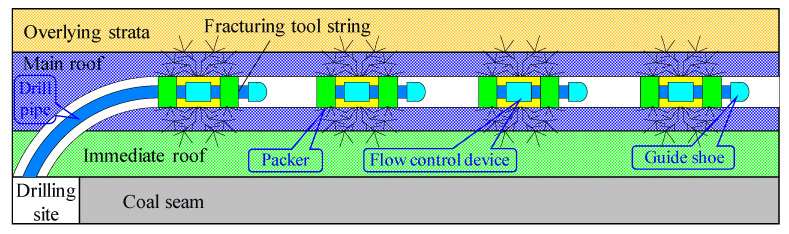
Sectional schematic of staged hydraulic fracturing using directional long boreholes.

Because of the large strike length of the LW2803, the spatial relationships between different advancing positions and the adjacent goaf, roadways, and coal pillars vary considerably. As a result, the degree of mining disturbance in the roof ahead of the working face and the characteristics of microseismic activity differ among different regions. If uniform borehole parameters are used throughout the entire working face, the pressure-relief requirements of different zones cannot be adequately satisfied. Therefore, zonal hydraulic fracturing is necessary according to the advancing condition of the working face and the distribution of microseismic activity. The staged hydraulic fracturing scheme using directional long boreholes was designed by comprehensively considering the geological structure at the construction site, rockburst manifestations in adjacent working faces, construction objectives, and roadway excavation conditions. Based on the strike length of the working face, the excavation plans of the two gate roads, and the designed borehole depth, the LW2803 was divided into four zones. From the open-off cut side, these zones were designated as zones I, II, III, and IV, respectively, as shown in Figure 1. Staged hydraulic fracturing using directional long boreholes was then carried out in different zones. To achieve staged fracturing within the target strata, directional long boreholes equipped with packers and fracturing tool strings were used. During fracturing, the packers isolated local sections of the borehole, and high-pressure water was injected into the predetermined horizon. This allowed fractures to preferentially propagate within the hard roof strata, thereby weakening their integrity and overall bearing capacity. A sectional schematic of the staged hydraulic fracturing process using directional long boreholes is shown in Figure 2.

### 2.2. Characteristics of Microseismic Distribution

**Figure 3:**
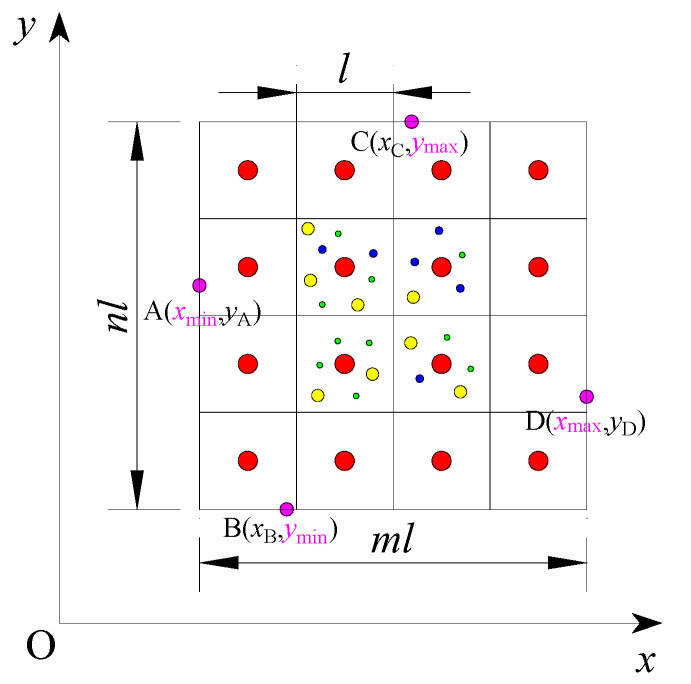
Schematic diagram of gridded statistics for microseismic events.

A large number of microseismic events were recorded during the mining of the working face. Directly projecting all events onto a plan view can reflect the general distribution range of microseismic activity, but events with different energy levels tend to overlap, making it difficult to identify the zones where microseismic energy is concentrated. To characterize the spatial clustering of microseismic events, a gridded statistical method was used in this study. The study area was divided into regular grids, and the total energy of microseismic events within each grid was calculated to represent the level of microseismic energy release in that grid. If the grid in the *i*th row and *j*th column contains *N* microseismic events, the microseismic energy in this grid can be expressed as

(1) $$E_{ij}=\sum_{k=1}^{N}E_{k}$$

where *E_ij_* is the energy of the grid in the *i*th row and *j*th column, and *E_k_* is the energy of the *k*th microseismic event in the corresponding grid. Because the energy of microseismic events spans several orders of magnitude, directly using the original energy values to plot the contour map would cause a few high-energy events to dominate the visualization, making the spatial differences among low-energy events difficult to distinguish. Therefore, the gridded energy was logarithmically transformed to obtain the corresponding energy level:

(2) $$e_{ij}=\log_{10}(E_{ij})=\log_{10}(\sum_{k=1}^{N}E_{k})$$

where *e_ij_* is the energy level of the grid in the *i*th row and *j*th column. By using the energy level, microseismic energies of different orders of magnitude can be compared on the same scale, which facilitates the identification of relatively concentrated microseismic energy zones within the working face. A schematic diagram of the gridded statistics for microseismic events is shown in Figure 3. Different colored spheres represent microseismic events with different energy levels. The red sphere located at the center of each grid indicates the energy level of that grid, while the purple spheres denote the boundary microseismic events used in the calculation, i.e., the events with the maximum and minimum coordinate values in each direction.

**Figure 4:**
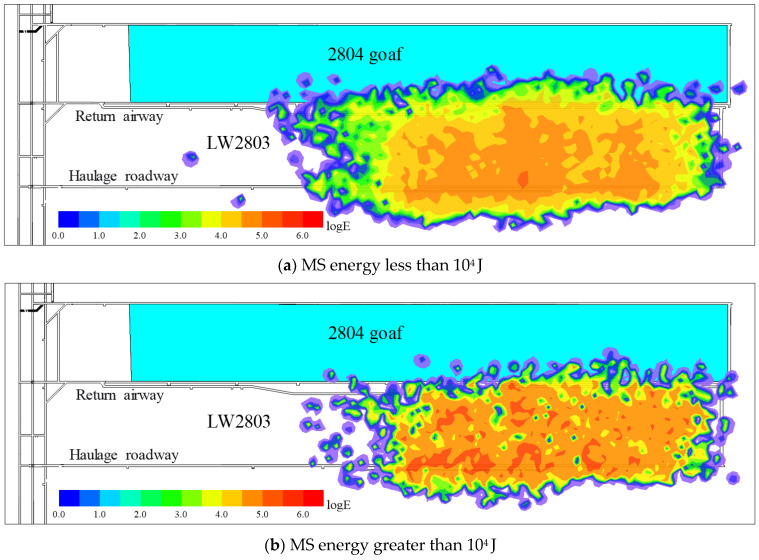
Spatial distribution of microseismic events with different energy levels.

**Figure 5:**
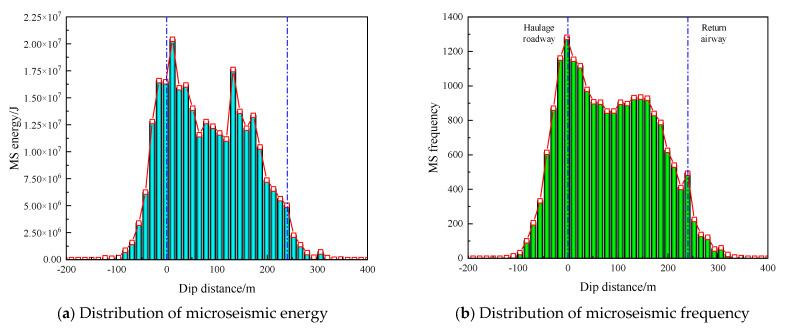
Distribution of microseismicity along the dip direction of the working face.

Based on the microseismic events monitored during the mining of the LW2803 from February 2025 to October 2025, spatial distribution maps of events with different energy levels were plotted. The variations in microseismic energy and frequency along the dip direction of the working face were further calculated, as shown in Figure 4 and Figure 5. To compare the spatial differences among events of different energy levels, 10^4^ J was used as the threshold to divide the events into low-energy events and relatively high-energy events.

Figure 4 shows that, after hydraulic fracturing, microseismic events in the LW2803 exhibit a markedly non-uniform spatial distribution. Low-energy events are distributed over a relatively wide range and show a continuous distribution along the strike direction of the working face. By contrast, high-energy events are more spatially confined and display stronger heterogeneity. For a goaf-side working face, overburden activity and lateral stress adjustment near the goaf commonly cause microseismic activity to concentrate on the goaf side. However, Figure 4 shows that neither high-energy nor low-energy events are concentrated toward the 2804 goaf. Instead, they are generally biased toward the haulage roadway side, indicating that the dominant zone of microseismic activity after hydraulic fracturing is mainly located on the side of the working face close to the haulage roadway.

Figure 5 further presents the statistical results of microseismic energy and frequency along the dip direction of the working face. Both the microseismic energy and frequency show clear skewed distributions in the dip direction. The high-value zones of the two curves are mainly located near the haulage roadway and its adjacent area, and then generally decrease or fluctuate downward toward the ventilation roadway. The microseismic energy curve reaches its main peak near the haulage roadway and shows secondary peaks at several internal positions of the working face, indicating several zones of relatively concentrated energy release. The microseismic frequency curve also peaks near the haulage roadway and remains relatively high within the working face, indicating that microseismic events occur more densely in this area.

Taken together, Figure 4 and Figure 5 show that, after hydraulic fracturing, microseismic activity is mainly concentrated within the mining-influenced area of the LW2803 and is generally biased toward the haulage roadway side. Low-energy microseismic events are widely distributed, whereas high-energy events are relatively concentrated. The high-value zones of microseismic energy and frequency show a certain spatial consistency, both gradually weakening from the haulage roadway side toward the ventilation roadway side. These results indicate that the post-fracturing microseismic distribution has an evident spatial redistribution characteristic, with the dominant microseismic activity zone shifting from the goaf side toward the haulage roadway side.

## 3. Microseismic-Driven Numerical Modeling Method for Hydraulic Fracturing-Induced Damage

### 3.1. Representation Method for Hydraulic Fracturing

To simulate the effect of hydraulic fracturing on the roof coal-rock mass in FLAC3D, microseismic events induced by hydraulic fracturing were introduced into the numerical model before the mining of the working face. The Benioff strain and damage variable of each numerical zone were calculated from the microseismic energy, thereby equivalently representing the damage weakening effect of hydraulic fracturing on the rock strata. This treatment was used to analyze the influence of hydraulic fracturing on the distribution of abutment pressure ahead of the working face.

**Figure 6:**
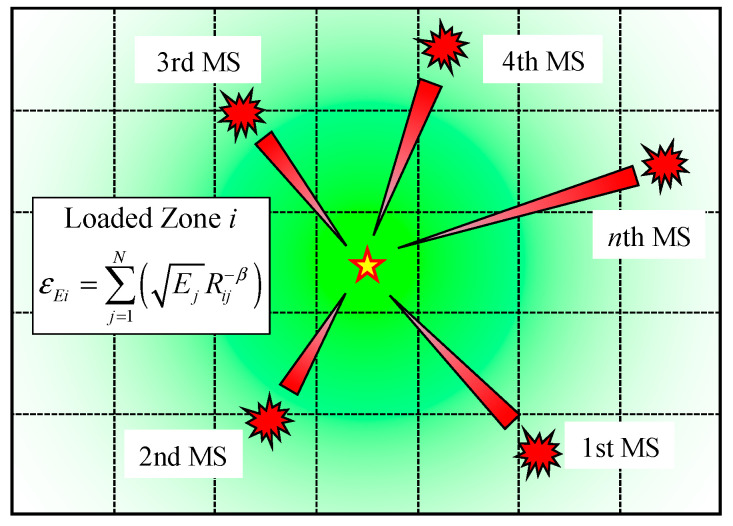
Schematic diagram for calculating the cumulative attenuated energy of numerical zone *i*.

During microseismic wave propagation, both the particle vibration velocity and the transmitted energy exhibit negative exponential attenuation. This attenuation is mainly caused by wavefront spreading and absorption by discontinuities in the rock mass. The propagation of peak particle velocity and energy can be expressed as [21,22]

(3) $$v_{pp}=v_{pp0}\cdot R^{-\alpha }$$

(4) $$E=E_{0}\cdot R^{-\beta }$$

where *v_pp_* is the attenuated peak particle velocity at a propagation distance *r*, *v_pp_*_0_ is the initial peak particle velocity at the source, *E* is the attenuated microseismic energy, *E*_0_ is the source energy of the microseismic event, and *α* and *β* are attenuation coefficients related to the propagation medium. According to previous studies, the rebound increment of elastic strain energy associated with a microseismic event is proportional to the square root of its released energy. The approximate relationship between peak particle velocity and the square root of microseismic energy is adopted under the following assumptions: the monitored events occur within the same mining area, the propagation medium and monitoring-system response are relatively consistent, and the radiated wave energy can be approximately related to the square of particle vibration velocity. Under these conditions, *v_pp_* can be used as an engineering indicator of dynamic-disturbance intensity and is approximately proportional to the square root of microseismic energy. Therefore, the relationship among microseismic energy, peak particle velocity, and strain can be expressed as [23,24]

(5) $$v_{pp}\propto \varepsilon_{E}\propto \sqrt{E}$$

where *ε_E_* denotes the Benioff strain associated with the microseismic energy. Accordingly, in FLAC3D, based on Equations (3)–(5), the Benioff strain of numerical zone *i* can be described using the attenuated energy derived from all hydraulic fracturing-induced microseismic events. Specifically, the Benioff strain acting on each numerical zone can be calculated by a superposition method [24]:

(6) $$\varepsilon_{Ei}=\sum_{j=1}^{N}\left(\sqrt{E_{j}}R_{ij}^{-\alpha /2}\right)=\sum_{j=1}^{N}\left(\sqrt{E_{j}}R_{ij}^{-\beta }\right)$$

where *ε_Ei_* is the cumulative Benioff strain of zone *i*, *E_j_* is the source energy of the *j*th microseismic event, *r_ij_* is the distance between zone *i* and the *j*th microseismic source, and *n* is the total number of hydraulic fracturing-induced microseismic events. The calculation process of the cumulative attenuated energy acting on numerical zone i is illustrated in Figure 6. Each hydraulic-fracturing-induced microseismic event contributes to the cumulative Benioff strain of the target zone according to its source energy and propagation distance.

Rock is a typical heterogeneous material containing various internal defects. These defects differ markedly in mechanical properties and are spatially non-uniform. During loading, the heterogeneity of the rock has a significant influence on stress distribution, deformation behavior, and damage failure. To characterize this heterogeneity, the rock mass is divided into a set of mesoscopic elements, and the mechanical parameters of these elements are assumed to obey a Weibull distribution. The probability density function is expressed as

(7) $$P\left(\varepsilon^{*}\right)=\frac{m}{F}{\left(\frac{\varepsilon^{*}}{\varepsilon_{F}}\right)}^{m-1}exp\left[-{\left(\frac{\varepsilon^{*}}{\varepsilon_{F}}\right)}^{m}\right]$$

where ε^*^ is the mesoscopic strength variable, which may represent elastic modulus, tensile strength, cohesion, strain, or deformation energy; *m* and *ε_F_* are the shape and scale parameters of the Weibull distribution, respectively. When Equation (7) is used to describe material heterogeneity, *m* is also referred to as the heterogeneity parameter. It reflects the concentration degree of mesoscopic strength distribution, or equivalently, the brittleness of the coal-rock material. A larger *m* indicates a more homogeneous material and a higher degree of brittleness. The parameter *ε_F_* represents the mean value of the mechanical variable ε^*^.

In this study, the damage probability is assumed to be related to strain energy and to follow a Weibull distribution. That is, a larger amount of deformation energy corresponds to a greater number of damaged mesoscopic elements. On this basis, under a given deformation-energy condition, the damage variable *D* can be calculated as the ratio of the number of damaged mesoscopic elements *N_d_* to the total number of mesoscopic elements *N*. The number of damaged mesoscopic elements within an arbitrary interval [*ε_E_*, *ε_E_* + *dε_E_*] is *NP*(*ε_E_*)*dε_E_*. When the strain energy reaches *ε_E_*, the number of damaged mesoscopic elements *N_d_* can be expressed as

(8) $$N_{d}\left(\varepsilon_{E}\right)=\int_{0}^{\varepsilon_{E}}NP\left(\varepsilon_{E}\right)d\varepsilon_{E}=N\left[1-exp\left(-\frac{\varepsilon_{E}}{\varepsilon_{F}}\right)\right]$$

Substituting the Weibull probability density function into the integral expression for the number of damaged mesoscopic elements and combining it with *D* = *N_d_*/*N*, the damage variable can be obtained as [25,26]

(9) $$D=1-exp\left(-\frac{\varepsilon_{E}}{\varepsilon_{F}}\right)$$

Equation (9) describes the internal damage degree of the rock mass. When *D* = 0, the rock mass is intact and undamaged; when *D* = 1, the rock mass is completely damaged. In Equation (9), *ε_F_* is the average cumulative Benioff strain, which can be determined by assuming that the critical damage value *D_c_* corresponds to the maximum cumulative Benioff strain (max{*ε_Ei_*}):

(10) $$\varepsilon_{F}=-\frac{\max\left\{\varepsilon_{Ei}\right\}}{\ln\left(1-D_{c}\right)}$$

where max{*ε_Ei_*} is the maximum cumulative Benioff strain in the model, and *D_c_* is the damage variable corresponding to the complete-damage state. In this study, *D_c_* is taken as 0.95.

**Figure 7:**
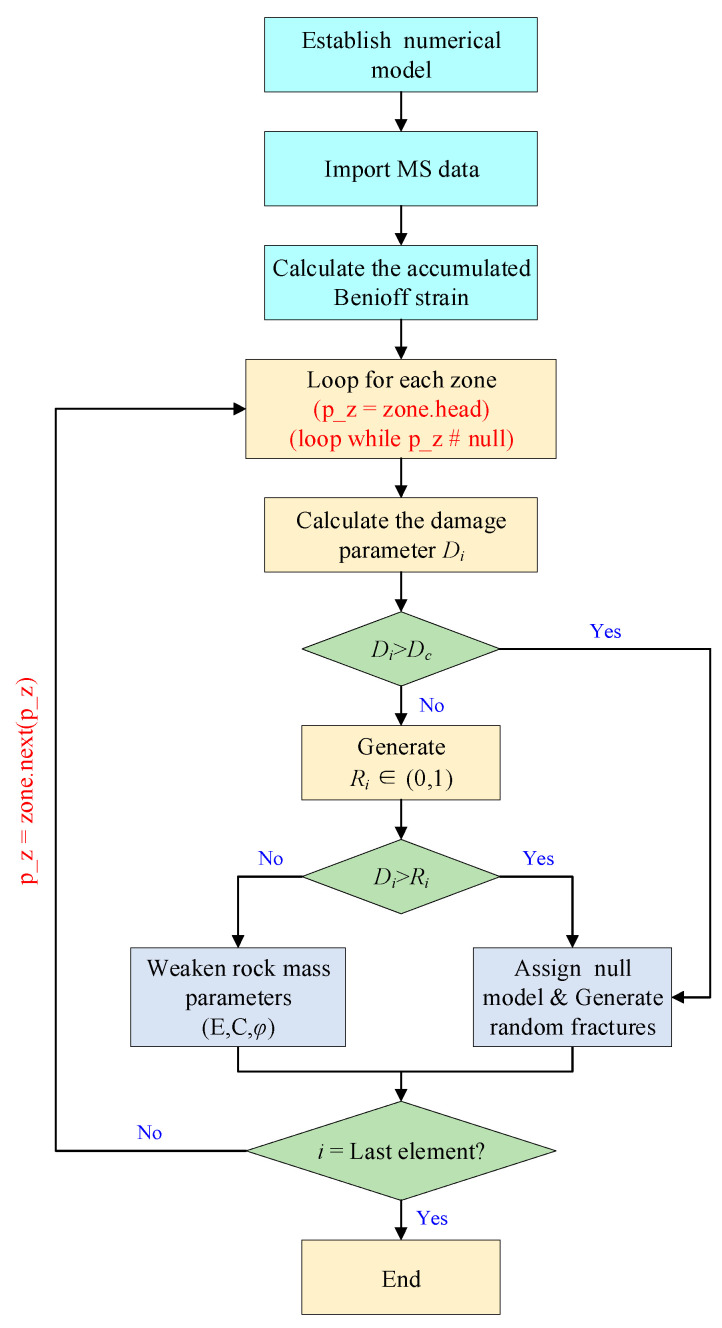
Flowchart of the numerical representation of hydraulic fracturing (The red text represents the FISH language used in FLAC3D).

Rock damage reduces the elastic modulus, cohesion, and internal friction angle of the material [27]. However, the quantitative relationship between the degradation degrees of cohesion, internal friction angle, and elastic modulus is difficult to determine. Therefore, the elastic modulus, cohesion, and internal friction angle are assumed to have the same damage degree. The damage variable of each numerical zone is used to weaken the rock-mass parameters according to Equations (11)–(13), thereby equivalently representing the damage weakening effect induced by hydraulic fracturing [28,29]:

(11) $$C_{i}=C_{i\_0}\left(1-D_{i}\right)$$

(12) $$E_{i}=E_{i\_0}\left(1-D_{i}\right)$$

(13) $$\varphi_{i}=arctan\left(tan\left(\varphi_{i0}\right)\left(1-D_{i}\right)\right)$$

where *C_i_*, *E_i_*, and *φ_i_* are the cohesion, elastic modulus, and internal friction angle of zone *i* after weakening, respectively; *C_i_*_0_, *E_i0_*, and *φ_i_*_0_ are the corresponding initial values before weakening; and *D_i_* is the damage variable of zone *i*. Meanwhile, a random number *R_i_* between 0 and 1 is generated for each numerical zone and compared with the corresponding damage variable *D_i_*. If *D_i_* > *R_i_*, the zone is regarded as a significantly fractured zone and is assigned the null model. This treatment is used to equivalently represent the formation of locally connected fractures or severely damaged zones after hydraulic fracturing. The overall simulation procedure is shown in Figure 7.

### 3.2. Numerical Model Setup

**Figure 8:**
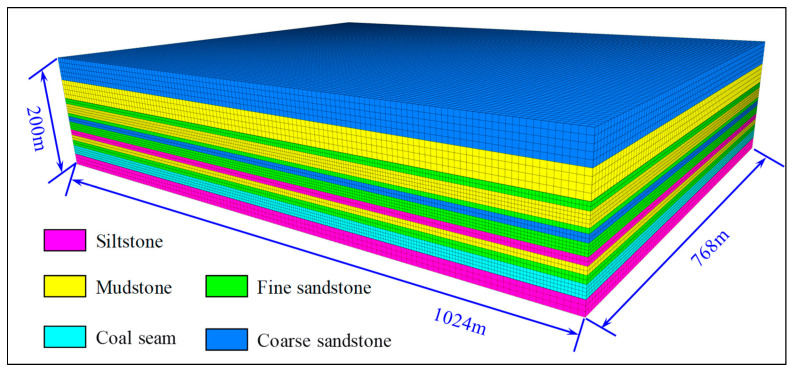
FLAC3D numerical model.

A simplified numerical model was established using FLAC3D 6.0 according to the geological conditions of Hetaoyu Coal Mine and the K31 borehole data, as shown in Figure 8. The model dimensions were 768 m × 1024 m × 200 m in the X, Y, and Z directions, respectively. The model domain included both the LW2804 and LW2803. The working face width and burial depth were set according to the actual field parameters. In the model, LW2804 was treated as a previously mined working face, whereas LW2803 was treated as the currently mined working face. This configuration was used to analyze the differences in stress and elastic strain energy distributions in the coal-rock mass before and after hydraulic fracturing under goaf-side conditions. All rock strata in the model were represented using the Mohr–Coulomb elastoplastic constitutive model. The physical and mechanical parameters of the coal-rock mass are listed in Table 1.

The vertical displacement was constrained at the bottom boundary of the model, and the normal displacement was constrained at the four lateral boundaries. The top boundary was set as a free surface. The initial stress field was generated by both self-weight stress and horizontal tectonic stress. The vertical stress was applied according to the self-weight of the overlying strata, while the horizontal stress was set to 1.2 times the vertical stress according to in situ stress measurement results. After the model reached initial equilibrium, the 2804 goaf was first formed, and the subsequent advance of the LW2803 was then simulated. To avoid boundary effects, the following analysis focused mainly on the coal-rock mass ahead of the LW2803, near the 2804 goaf-side boundary, and within the hydraulic fracturing-affected zone.

To compare the effect of hydraulic fracturing, two calculation cases were designed.

1. Normal mining case: Hydraulic fracturing-induced damage was not introduced, and only the normal mining process of the 2803 goaf-side working face was simulated. This case was used to obtain the stress and elastic strain energy distributions ahead of the working face under non-fractured conditions.
2. Hydraulic fracturing case: Before mining of the LW2803, hydraulic fracturing-induced damage was introduced into the target hard roof strata according to the microseismic energy–Benioff strain–Weibull damage mapping relationship established in Section 3.1. This treatment was used to represent locally connected fractures or strongly weakened zones after hydraulic fracturing. Except for the treatment of fracturing-induced damage, the model dimensions, boundary conditions, rock-mass parameters, initial stress field, and mining process were kept identical in the two cases.

For reasons of computational efficiency and article length, the analysis focused on the No. 1 drilling site as a representative example to examine the stress and energy distributions before and after hydraulic fracturing. The advance of the working face was simulated by stepwise excavation. After equilibrium was reached at each advancing stage, the vertical stress, elastic strain energy, and damage distribution were extracted for both the normal mining case and the hydraulic fracturing case. To further analyze the changes in the loading and energy-storage states within the coal seam, a monitoring line was arranged along the dip direction of the working face inside the coal seam, and the stress and elastic strain energy curves at different advancing stages were extracted. This monitoring line passed through the coal mass ahead of the working face, the solid-coal side, and the goaf-side adjacent area, enabling comparison of the spatial changes in high-stress and high-elastic-strain-energy zones in the coal seam before and after hydraulic fracturing.

The numerical zones in the model satisfy the Mohr–Coulomb yield criterion. The elastic strain energy *U* stored in the coal-rock mass can be calculated as

(14) $$U=\frac{1}{2E}\left[\sigma_{1}^{2}+\sigma_{2}^{2}+\sigma_{3}^{2}-2\mu \left(\sigma_{1}\sigma_{2}+\sigma_{1}\sigma_{3}+\sigma_{2}\sigma_{3}\right)\right]$$

where *E* is the elastic modulus of the coal-rock mass; *σ*_1_, *σ*_2_, and *σ*_3_ are the maximum, intermediate, and minimum principal stresses, respectively; and *μ* is Poisson’s ratio.

### 3.3. Parameter Calibration

**Figure 9:**
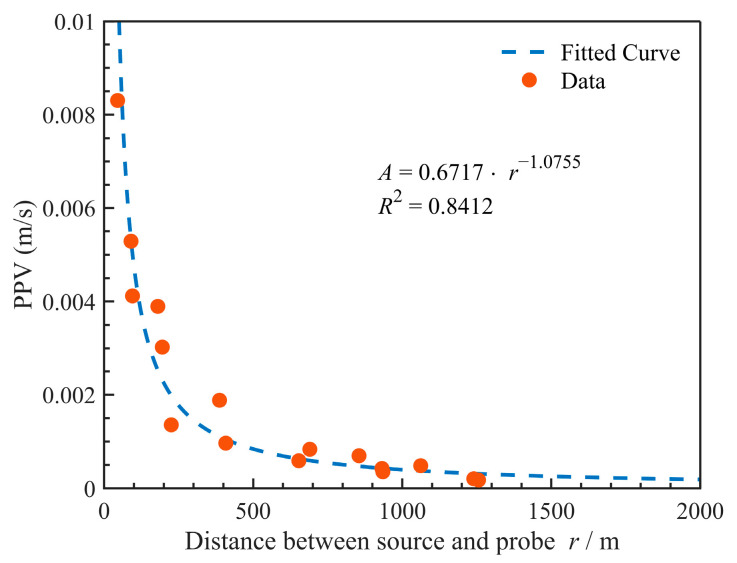
Fitted curve of PPV attenuation.

To determine the energy attenuation coefficient β, the PPV attenuation coefficient α is first calibrated based on the field microseismic monitoring data from Hetaoyu Coal Mine. The microseismic event energy E is obtained by the field microseismic monitoring system via waveform inversion, whereas α is determined through least-squares fitting using the source-geophone distance and peak particle velocity data. The fitting results are shown in Figure 9, indicating that α = 1.0755, and thus β = 2α = 2.1510.

In this study, the heterogeneity parameter m can be analytically determined from the laboratory uniaxial compressive stress–strain curve. Based on the boundary condition that the first derivative of stress with respect to strain equals zero at the peak stress point (d*σ*/d*ε* = 0), the parameter *m* is explicitly calculated as follows [30]:

(15) $$m=\frac{1}{\ln\left(\frac{E\varepsilon_{c}}{\sigma_{c}}\right)}$$

where E, *σ*_c_, and *ε*_c_ denote the elastic modulus, uniaxial compressive strength, and peak strain of the rock specimen, respectively. This method ensures that the selection of parameter m is physically grounded and objectively calibrated by experimental data. Based on the laboratory-tested stress–strain curves of the rock specimens collected from Hetaoyu Coal Mine, the Weibull parameter *m* was determined to be 2.67.

## 4. Energy Redistribution Mechanism After Hydraulic Fracturing

### 4.1. Spatial Distribution of the Heterogeneous Damage Zone

**Figure 10:**
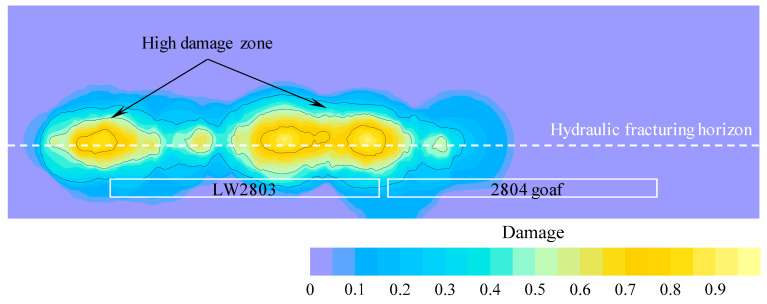
Damage contour ahead of the working face after an advance of 200 m.

The spatial morphology of the fracturing-induced damage zone provides the basis for analyzing the redistribution of the stress field and elastic strain energy field after hydraulic fracturing. Based on the microseismic-driven damage characterization method established in Section 3.1, the damage distribution in the target strata and adjacent surrounding rock after hydraulic fracturing was extracted, as shown in Figure 10.

Figure 10 shows that an evident high-damage zone forms in the overlying strata ahead of the working face. The high-damage zone is mainly distributed above the area ahead of the LW2803 and near the 2804 goaf-side area, showing good spatial correspondence with the designed fracturing horizon. This result indicates that directional long-borehole hydraulic fracturing forms a heterogeneous damage-weakened zone within the target hard roof. In terms of damage morphology, the high-damage zone is not uniformly and continuously distributed, but appears as multiple high-value patches that are interconnected and locally connected. This suggests that the formation of fracturing-induced damage is jointly controlled by rock heterogeneity, bedding structures, and mining-induced stress disturbance. Although the damage contour cannot be directly equated with the actual geometry of hydraulic fractures, the development of the high-damage zone in the overlying strata adjacent to the 2804 goaf, together with the formation of a low-stress zone on the goaf side after fracturing, indicates that the hydraulic fractures and the associated induced damage tend to develop toward the goaf-adjacent area.

The development of the high-damage zone in the overlying strata near the 2804 goaf indicates that hydraulic fracturing has a strong modifying effect on the roof structure on the goaf side. This heterogeneous damage zone reduces the equivalent stiffness and local bearing capacity of the target strata, thereby providing the structural basis for subsequent stress-path adjustment and elastic strain energy redistribution during mining.

### 4.2. Spatial Response Characteristics of Stress and Elastic Strain Energy

**Figure 11:**
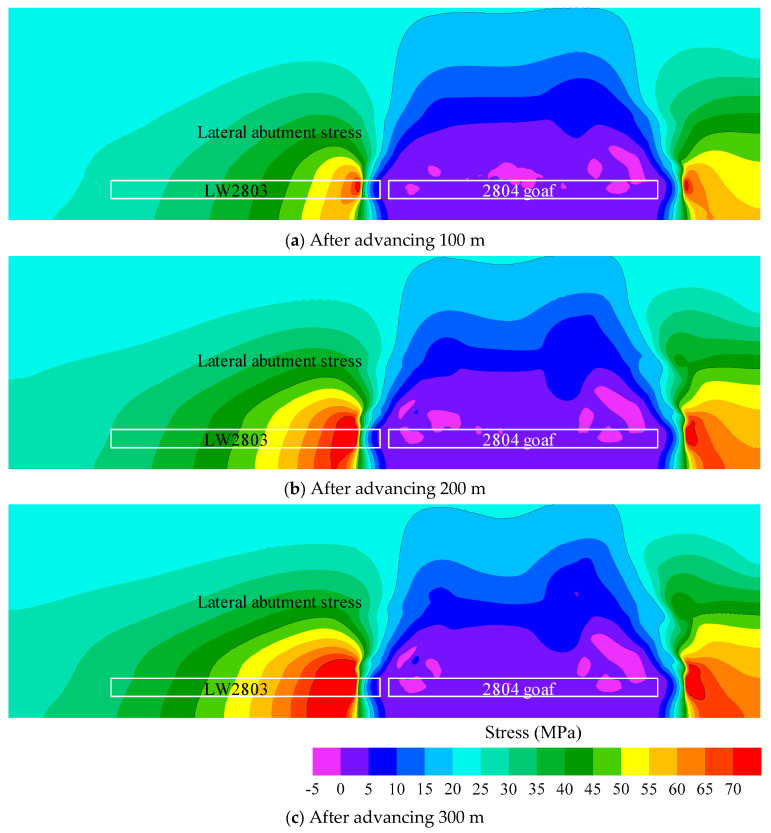
Stress distribution ahead of the working face under normal mining conditions.

**Figure 12:**
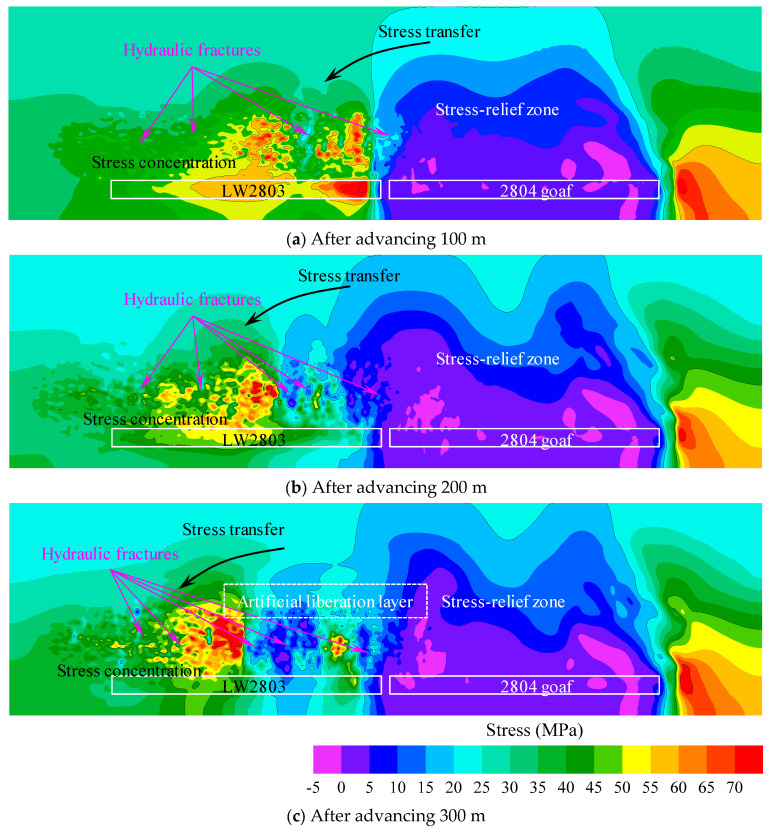
Stress distribution ahead of the working face under hydraulic fracturing conditions.

**Figure 13:**
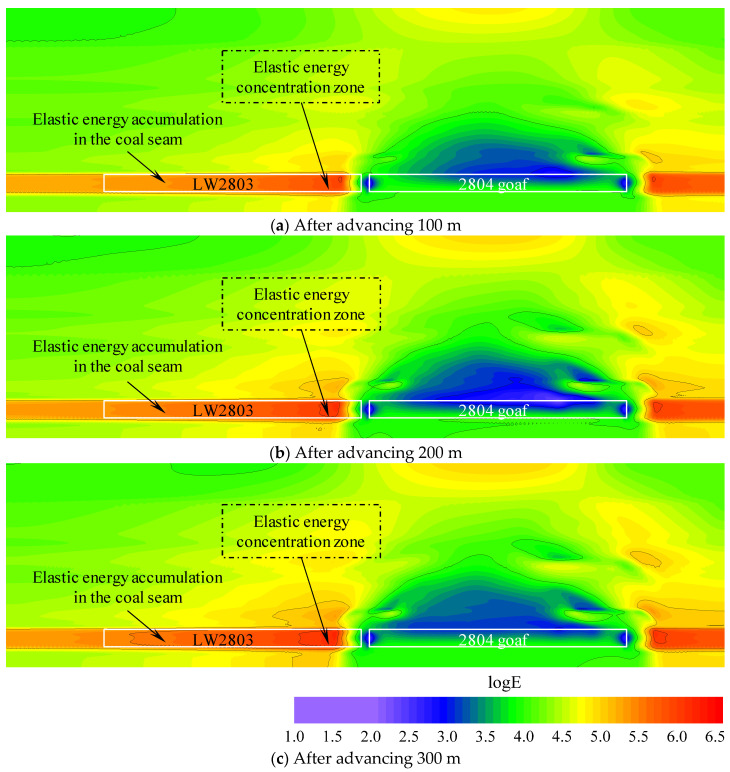
Elastic strain energy distribution ahead of the working face under normal mining conditions.

**Figure 14:**
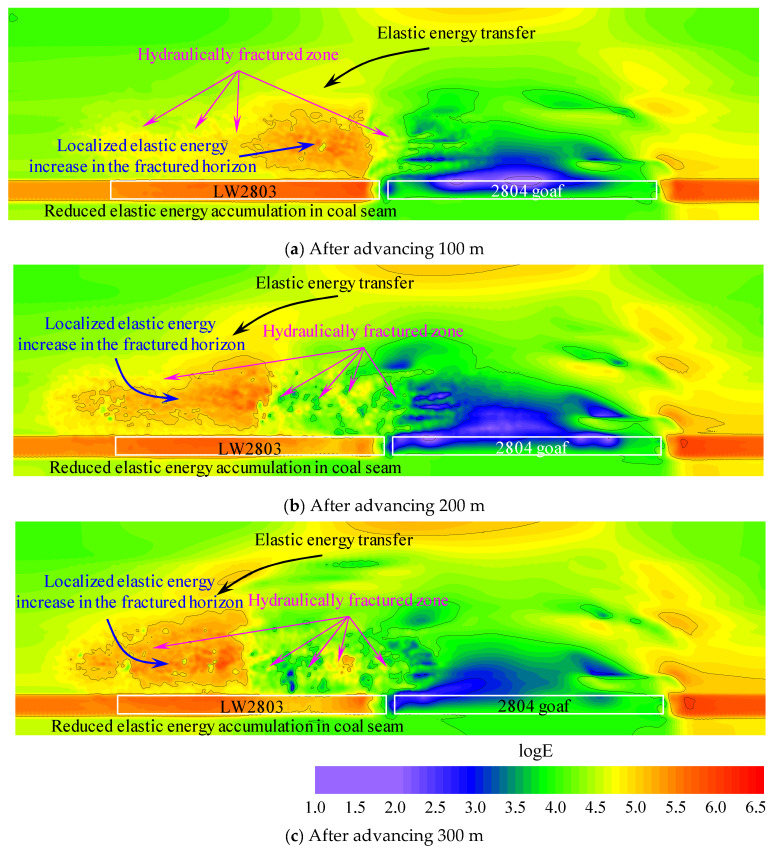
Elastic strain energy distribution ahead of the working face under hydraulic fracturing conditions.

After clarifying the spatial distribution of the fracturing-induced damage zone, the evolution characteristics of the stress field and elastic strain energy field ahead of the working face were further compared under normal mining and hydraulic fracturing conditions. Figure 11 and Figure 12 show the stress distributions after advances of 100 m, 200 m, and 300 m under normal mining and hydraulic fracturing conditions, respectively. Figure 13 and Figure 14 present the corresponding elastic strain energy distributions.

Figure 11 shows that, under non-fractured conditions, an evident lateral abutment stress concentration zone forms ahead of the LW2803. The high-stress zone is mainly distributed in the coal-rock mass ahead of the working face and near the boundary of the 2804 goaf, and it migrates forward as the working face advances. From an advance of 100 m to 300 m, the high-stress zone maintains strong spatial continuity, indicating that the overlying hard roof still has a strong integral bearing capacity under non-fractured conditions. Consequently, mining-induced load can be continuously transferred and concentrated toward the area ahead of the working face and the goaf-side region.

Compared with the normal mining condition, the stress field ahead of the working face changes markedly after hydraulic fracturing, as shown in Figure 12. Local stress perturbation occurs within and near the boundary of the fracturing-affected zone, reflecting stiffness contrast and load redistribution between the damaged zone and the residual intact rock mass. However, from the overall distribution, the continuity of the high-stress zone is clearly weakened, and the continuous high-stress range in the coal-rock mass ahead of the working face is reduced. This indicates that hydraulic fracturing weakens the continuous concentrated loading state of the coal-rock mass.

Figure 13 shows that, under non-fractured conditions, a distinct elastic strain energy concentration zone exists in the coal seam and adjacent surrounding rock ahead of the working face. As mining proceeds, the high-energy zone migrates forward and continues to accumulate. This distribution corresponds well to the lateral abutment stress concentration zone under non-fractured conditions, indicating that mining-induced load concentration not only increases local stress but also promotes elastic strain energy accumulation in the coal-rock mass.

After hydraulic fracturing, the elastic strain energy field ahead of the working face exhibits obvious redistribution, as shown in Figure 14. The extent of the high elastic strain energy zone in the coal seam and adjacent surrounding rock decreases, and its continuity is weakened, indicating that the continuous energy-storage capacity of the coal-rock mass is suppressed after fracturing. Meanwhile, local increases in elastic strain energy may occur near the fractured horizon, the boundary of the damage zone, or residual rock bridges. These local increases mainly reflect load-path adjustment and local reorganization of the bearing structure after hydraulic fracturing, rather than the reformation of a continuous high-energy-storage state in the coal seam.

The combined stress and elastic strain energy distributions show that, under non-fractured conditions, the high-stress zone and high elastic strain energy zone are strongly consistent in space. This indicates that, when the hard roof maintains an intact bearing structure, the coal-rock mass ahead of the working face remains in a continuous loading-continuous energy-storage state. After hydraulic fracturing, the continuity of both the high-stress zone and the high elastic strain energy zone is weakened. This demonstrates that fracturing-induced damage does not simply cause a global reduction in stress or energy. Instead, it disrupts the original continuous load-transfer and energy-storage structures, causing the stress and elastic strain energy responses to change from continuous concentration to localized and dispersed distributions.

**Figure 15:**
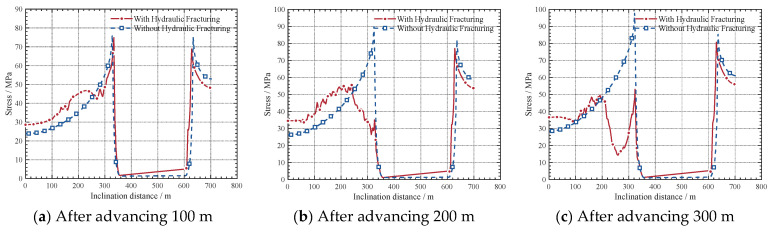
Coal-seam stress curves at different advancing stages.

**Figure 16:**
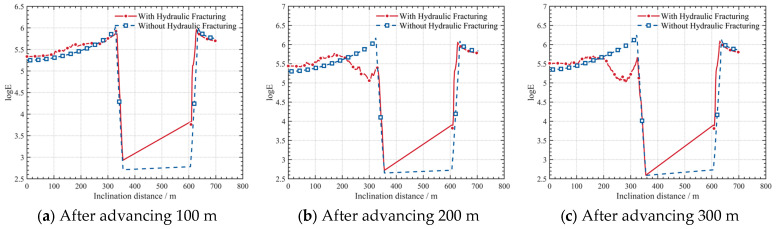
Coal-seam elastic strain energy curves at different advancing stages.

To further quantify the changes in the loading and energy-storage states of the coal seam, a typical monitoring line was arranged in the coal seam. Stress and elastic strain energy curves were extracted for the normal mining and hydraulic fracturing conditions after working face advances of 100 m, 200 m, and 300 m, as shown in Figure 15 and Figure 16.

Figure 15 shows that, under normal mining conditions, the coal-seam stress along the monitoring line exhibits a clearly non-uniform distribution. High stress is mainly concentrated ahead of the working face and near the 2804 goaf boundary. After hydraulic fracturing, the difference between the stress curves before and after fracturing is relatively small when the working face advances 100 m. However, at advances of 200 m and 300 m, the stress in the middle part of the coal seam and in some sections ahead of the working face is clearly lower than that under normal mining conditions. The range of the continuous high-stress zone is also reduced. This indicates that the coupling effect between the fracturing-induced damage zone and mining disturbance gradually strengthens as the working face advances.

The elastic strain energy curves correspond well to the stress curves, as shown in Figure 16. Under normal mining conditions, the elastic strain energy in the coal seam remains at a high level ahead of the working face and near the 2804 goaf boundary. In particular, after advances of 200 m and 300 m, the elastic strain energy curves show a relatively continuous increasing trend ahead of the working face, reflecting the continuous energy-storage behavior of the coal seam under mining-induced loading. After hydraulic fracturing, the coal-seam elastic strain energy curves generally show weakened and more dispersed characteristics, and the elastic strain energy level decreases significantly in some sections. This indicates that the continuous energy-storage state of the coal seam is weakened after fracturing.

**Figure 17:**
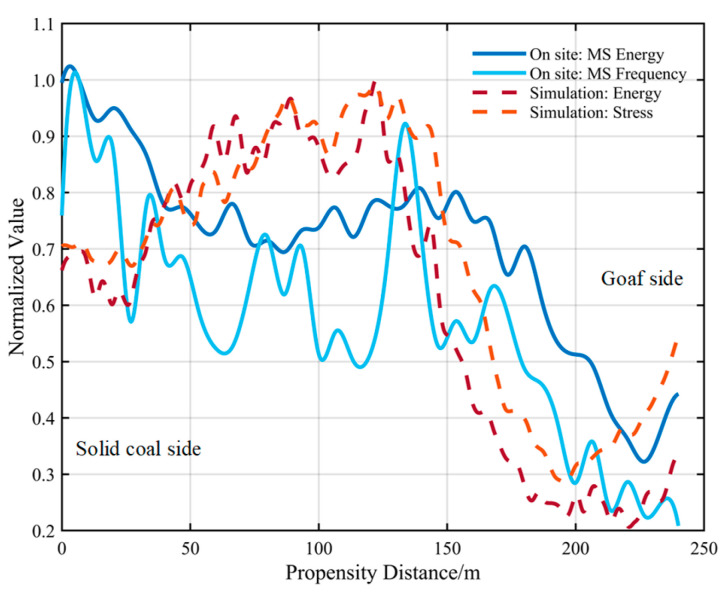
Normalized comparison between field microseismic data and numerical simulation results.

To further quantify the spatial consistency between the numerical simulation results and field microseismic observations, the field microseismic energy, microseismic frequency, and the simulated stress and elastic strain energy are normalized along the dip direction of the working face and integrated into a unified spatial coordinate system for comparison, as shown in Figure 17.

As shown in Figure 17, the field microseismic energy and microseismic frequency exhibit prominent high values in the region adjacent to the solid-coal side and decrease overall along the dip direction. Similarly, the normalized curves of stress and elastic strain energy from the numerical simulation maintain a relatively high response in the corresponding region, followed by a downward trend. The two display good consistency in both the locations of the high-value zones and their spatial variation trends, demonstrating that the numerical simulation can effectively reflect the primary characteristic of the mining-induced stress–energy response concentrating toward the solid-coal side after hydraulic fracturing.

The combined contour, monitoring line, and normalized spatial comparison results show that the main function of hydraulic fracturing is not to simply eliminate local stress peaks, but to weaken the continuous concentrated loading and continuous energy-storage state of the coal seam. The synchronous changes in stress and elastic strain energy curves indicate that the fracturing-induced damage zone can reduce the capacity of the coal seam to sustain continuous concentrated loading and further suppress continuous elastic strain energy accumulation. These results provide stress–energy field evidence for the subsequent analysis of weakened goaf-side load-transfer and asymmetric energy redistribution.

### 4.3. Rockburst Prevention Mechanism Through Load Reduction and Energy Dissipation

**Figure 18:**
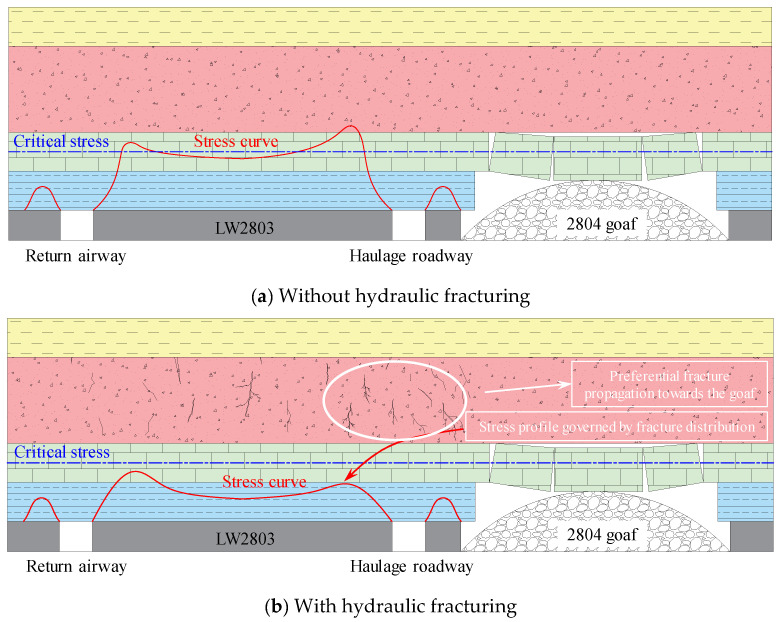
Schematic diagram of the hard-roof load-transfer mode and coal-seam energy-storage response before and after hydraulic fracturing.

Based on the comparative results of fracturing-induced damage distribution, stress field, and elastic strain energy field, the differences in the hard-roof load-transfer mode and coal-seam energy-storage response before and after hydraulic fracturing can be further conceptualized, as shown in Figure 18.

Under non-fractured conditions, the hard roof maintains strong integrity and continuous bearing capacity. After mining of the working face, mining-induced load can be transferred through the continuous roof structure toward the area ahead of the working face and the region adjacent to the 2804 goaf, causing continuous high-stress zones to form locally in the coal seam. Because elastic strain energy accumulation is controlled by the stress state, the high-stress zones also correspond to high elastic strain energy zones. As a result, the coal-rock mass remains in a continuous loading and continuous energy-storage state. This state provides the static-load and energy basis for subsequent strong ground pressure manifestations or dynamic-disturbance-induced failure.

After hydraulic fracturing, fractures induced by directional long boreholes propagate within the target hard roof and form a heterogeneous damage-weakened zone. Under the influence of the low-confinement boundary on the 2804 goaf side and mining-induced stress adjustment, the high-damage zone mainly develops in the overlying strata adjacent to the goaf. This damage-weakened zone reduces the equivalent stiffness and local bearing capacity of the target strata, weakening the conditions for continuous concentrated load-transfer from the goaf-side overburden to the coal seam. Consequently, mining-induced load-transfer changes from the pre-fracturing continuous concentrated mode to a dispersed mode jointly controlled by the damaged zone, residual rock bridges, and locally intact rock masses.

Under this adjustment of the load-transfer structure, the concentrated loading and continuous energy-storage capacity of the coal-rock mass on the goaf side are suppressed, and part of the residual mining-induced load is redistributed toward the solid-coal side and the roadway-adjacent region. As a result, the high-value zones of microseismic energy and frequency in the field show a biased distribution toward the haulage roadway side. This phenomenon does not mean that hydraulic fracturing increases the overall energy level. Rather, it indicates that the original continuous energy-storage path on the goaf side is disrupted after fracturing, causing the dominant zone of energy release to undergo spatial adjustment.

Therefore, the rockburst-control effect of directional long-borehole hydraulic fracturing should be understood as bearing-structure reconstruction, load-path adjustment, and disruption of the continuous energy-storage state under the control of a fracturing-induced heterogeneous damage zone. The action chain can be summarized as follows: hydraulic fracturing induces the formation of a heterogeneous damage-weakened zone; this zone cuts the continuous load-transfer structure of the hard roof; mining-induced load-transfer changes from concentrated transfer to dispersed transfer; elastic strain energy accumulation in the coal seam changes from continuous accumulation to local dispersion; and the high static-load and high-energy-storage state of the coal-rock mass ahead of the working face is finally weakened, thereby achieving rockburst prevention through load reduction and energy dissipation.

## 5. Discussion

### 5.1. Null Model Treatment and Mesh Dependence

Because null elements completely lose their load-bearing capacity, this treatment may locally enhance the unloading effect and cause abrupt stress redistribution in adjacent elements. Therefore, the results obtained using this method are more suitable for analyzing the overall redistribution of macroscopic fields after hydraulic fracturing at the stope scale, rather than for revealing detailed mechanical responses at the fracture scale.

In addition, the mesh size affects the spatial discretization of the strongly damaged zone and the characteristics of local stress redistribution. A smaller mesh size is beneficial for describing the fractured damage zone more precisely and weakening the stress-jump effect caused by a single null element, thereby improving numerical stability. However, for a large-scale three-dimensional stope model, the mesh size cannot be reduced indefinitely due to limitations in computational efficiency and model scale. Therefore, the mesh size adopted in this study represents an engineering compromise between numerical stability and computational efficiency. It can support the analysis of the overall evolution of the stress–energy field at the stope scale, but its influence on local stress peaks and the boundary morphology of strongly damaged zones still requires further quantitative evaluation.

In future work, comparative simulations with different mesh sizes will be conducted. In addition, a damage-parameter reduction method retaining residual strength and residual stiffness will be introduced to further reduce the local stress jumps and mesh dependency that may be caused by the complete nullification of strongly damaged elements, thereby improving the stability and rationality of the representation of hydraulic fracturing-induced damage zones.

### 5.2. Isotropic Damage Assumption

In this study, a scalar damage variable D is adopted for the equivalent characterization of hydraulic fracturing-induced damage, through which the elastic modulus, cohesion, and internal friction angle are synchronously degraded. Therefore, although this method can reflect the spatial heterogeneity of the fracturing-induced damage zone, it inherently belongs to an equivalent isotropic damage treatment. In reality, hydraulic fractures induced by directional long boreholes are jointly controlled by the borehole axis, bedding structures, and in situ stress orientations, typically exhibiting a preferred propagation direction; consequently, the degrees of stiffness and strength degradation may vary along different directions. The Weibull distribution employed in this paper is primarily used to describe the spatial heterogeneity of damage, which cannot fully characterize the mechanical anisotropy induced by fracture directionality.

Since this study primarily focuses on the macro-scale influence of the hydraulic fracturing damage zone on the overall redistribution of the stress and elastic strain energy fields at the stope scale, rather than single fracture propagation or localized crack-tip responses, the aforementioned equivalent treatment is sufficient to satisfy the analysis requirements for the macroscopic evolution laws. However, this simplification may smooth out the influence of the preferred fracture propagation direction on localized load-transfer paths, and potentially underestimate the localized unloading and stress redistribution characteristics along the direction of fracture interconnection. Future research can further incorporate directional fracture characterization or anisotropic damage models to enhance the capability of representing the directionality of directional hydraulic fracturing fractures and their corresponding mechanical effects.

### 5.3. Scope of Applicability

Taking the 2803 goaf-side working face in Hetaoyu Coal Mine as the engineering background, the hydraulic fracturing-based rockburst-control mechanism revealed in this study is established on the basis of the continuous bearing capacity of the hard roof, fracturing-induced damage weakening, and the redistribution of mining-induced stress and elastic strain energy. For working faces with similar conditions of hard-roof control, goaf-side mining, and mining-induced energy concentration, directional long-borehole hydraulic fracturing can weaken the structural integrity of key roof strata and alter the original continuous load-transfer paths, thereby reducing the concentration of localized high stress and high elastic strain energy.

It should be noted that the post-fracturing stress–energy redistribution characteristics are closely related to specific engineering and geological conditions. When the coal seam occurrence, burial depth, roof lithological combination, structural development, or goaf compaction characteristics change significantly, the breaking modes of the hard roof, the propagation morphology of fracturing-induced fractures, and the mining-induced load-transfer paths may also vary accordingly. Therefore, the conclusions of this paper primarily reflect the regulatory effects of directional long-borehole hydraulic fracturing on the roof bearing structure and the mining-induced stress–energy distribution under goaf-side hard-roof working face conditions. For working faces with substantial differences in geological structures, burial depths, roof lithologies, and goaf confinement states, the specific performance of this mechanism still needs further validation combined with site conditions. Subsequent research will integrate field microseismic monitoring, fracturing effect detection, and multi-scenario numerical simulations under diverse engineering conditions to further improve the applicability evaluation of this mechanism.

## 6. Conclusions

Taking the 2803 goaf-side working face in Hetaoyu Coal Mine as the engineering background, this study developed a hydraulic fracturing-induced damage characterization method based on field microseismic monitoring data and incorporated it into a FLAC3D numerical model. The evolution characteristics of the damage field, stress field, and elastic strain energy field ahead of the working face were compared under normal mining and hydraulic fracturing conditions. The main conclusions are as follows:

(1) After hydraulic fracturing, microseismic activity in the LW2803 shows a markedly non-uniform spatial distribution. Low-energy events are distributed over a relatively wide range, whereas high-energy events are more spatially concentrated. The high-value zones of microseismic energy and frequency are mainly located near the haulage roadway and its adjacent area, rather than being concentrated toward the 2804 goaf side. This indicates that the dominant energy release zone of the goaf-side working face is spatially adjusted after hydraulic fracturing.
(2) A hydraulic fracturing-induced damage characterization method based on “microseismic energy–Benioff strain–Weibull damage” mapping relationship is established. In this method, the energy of fracturing-induced microseismic events is converted into the cumulative Benioff strain of numerical zones to determine the corresponding rock damage variable. By degrading the elastic modulus, cohesion, and internal friction angle in the FLAC3D finite-difference model, the heterogeneous damage-weakened state of the roof can be appropriately represented, thereby reducing the subjectivity associated with manually predefined homogeneous weakened zones in conventional simulations.
(3) The numerical results show that, after hydraulic fracturing, a patchy and locally connected high-damage weakening zone forms in the target hard roof. The high-damage zone is mainly distributed ahead of the LW2803 and in the overlying strata adjacent to the 2804 goaf. This heterogeneous damage structure reduces the equivalent stiffness and local bearing capacity of the target strata, providing the structural basis for subsequent adjustment of the mining-induced load path.
(4) Under non-fractured conditions, continuous high-stress zones form ahead of the working face and near the 2804 goaf boundary. High elastic strain energy accumulation zones also occur in the coal seam and adjacent surrounding rock, showing good spatial correspondence with the high-stress zones. This indicates that, when the hard roof maintains an intact bearing structure, the coal-rock mass remains in a continuous loading-continuous energy-storage state. After hydraulic fracturing, the continuity of the high-stress and high elastic strain energy zones is synchronously weakened, and the stress and elastic strain energy responses of the coal seam change from continuous concentration to localized and dispersed distributions. This demonstrates that fracturing-induced damage can weaken the capacity of the coal-rock mass to sustain concentrated loading and continuous energy accumulation.
(5) Directional long-borehole hydraulic fracturing modifies the bearing structure of the hard roof through the formation of a heterogeneous damage-weakened zone. This process alters the mining-induced load-transfer path, shifting it from a continuous concentrated mode to a relatively dispersed mode, and changes the coal-seam elastic strain energy from continuous accumulation to localized dispersion. Consequently, the high static-load and high-energy-storage state ahead of the working face is effectively weakened, demonstrating that the prevention mechanism relies on bearing-structure modification and load-path adjustment under the control of the heterogeneous damage zone.

## Figures and Tables

**Table 1:** Physical and mechanical parameters of the coal and rock strata.

Lithology	Bulk Modulus (GPa)	Shear Modulus (GPa)	Cohesion (MPa)	Friction Angle (°)	Tensile Strength (MPa)	Density (kg/m^3^)
Mudstone	11.23	7.40	7.10	39.0	2.95	2540
Coarse sandstone	13.92	11.78	22.20	40.0	4.45	2440
Siltstone	12.84	9.24	12.50	39.0	3.52	2470
Fine sandstone	13.03	9.77	17.24	39.0	4.07	2470
Coal seam	1.36	1.14	4.80	30.0	2.26	1303

## Data Availability

The data presented in this study are available on request from the corresponding author.

## References

[1] Xie H.P. (2019). Research review of the state key research development program of China: Deep rock mechanics and mining theory. J. China Coal Soc..

[2] Yang J.X., Liu C.Y., Yu B., Lu Y., Yang Y. (2014). Impact effect caused by the fracture of thick and hard roof structures in a longwall face. J. China Univ. Min. Technol..

[3] He J., Dou L.M., Wang S.W., Shan C.H. (2017). Study on mechanism and types of hard roof inducing rock burst. J. Min. Saf. Eng..

[4] Yang J.X., Lu Y., Liu C.Y., Yang Y. (2013). Analysis on the rock failure and strata behavior characteristics under the condition of hard and thick roof. J. Min. Saf. Eng..

[5] Gao M.S., Xu D., He Y.L., Zhang Z.G., Yu X. (2022). Investigation on the near-far field effect of rock burst subject to the breakage of thick and hard overburden. J. Min. Saf. Eng..

[6] Shen W.L., Bai J.B., Wang X.Y., Yu Y. (2016). Response and control technology for entry loaded by mining abutment stress of a thick hard roof. Int. J. Rock Mech. Min. Sci..

[7] Tan Y., Wang Y., He M.C., Li H., Guo W.B., Li L.M., Liu W.D., Zhang S.P. (2025). Multi dimensional segmented hydraulic fracturing impact ground pressure control and loss reduction technology for deep buried thick coal seam hard roof. J. China Coal Soc..

[8] Lv H., Cheng Z., Xie F., Pan J.F., Liu F. (2024). Study on hydraulic fracturing prevention and control of rock burst in roof of deep extra-thick coal seam roadway group. Sci. Rep..

[9] Kang H., Jiang P., Feng Y., Gao F.Q., Zhang Z., Liu X.G. (2023). Application of Large-Scale Hydraulic Fracturing for Reducing Mining-Induced Stress and Microseismic Events: A Comprehensive Case Study. Rock Mech. Rock Eng..

[10] Ma Y.Z., Gao Y.T., Zhu S.T., Pan J.F., Xia K.W., Zhang X.F., Jiang F.X., Liu J.H., Wang B., Chen Y. (2024). Mechanism and evaluation method of rock burst prevention in coal minesusing surface hydraulic fracturing. J. China Coal Soc..

[11] Bian J., Liu A.X., Yang S., Lu Q., Jia B., Li F., Ma X., Gong S., Cai W. (2024). A Combined Method of Seismic Monitoring and Transient Electromagnetic Detection for the Evaluation of Hydraulic Fracturing Effect in Coal Burst Prevention. Sensors.

[12] Zhang H.W., Zhou C.J., He J., Yang Q.S., Ye Z.W., Zhang S.X., Rui X.S., Zhan L.Y., Ren B.H. (2025). A coupled simulation method of hydraulic fracturing for relieving mining pressureunder hard roof based on DEM method. J. China Coal Soc..

[13] Cao X., Wu S., He Q. (2024). Investigation into Influences of Hydraulic Fracturing for Hard Rock Weakening in Underground Mines. Appl. Sci..

[14] Yu B., Gao R., Xia B.W., Kuang T.J. (2021). Ground fracturing technology and application of hard roof in large space. J. China Coal Soc..

[15] Wang Y.J., Xu G., Chen F.B., Lu Y.B., Li Y., Sun X.B., Liu N. (2022). Mining pressure weakening mechanism by ground fracturing and fracturing evaluation of hard rock strata. J. Min. Strat. Control Eng..

[16] Pan J.F., Kang H.P., Yan Y.D., Ma X.H., Ma W.T., Lu C., Lv D.Z., Xu G., Feng M.H., Xia Y.X. (2023). The method, mechanism and application of preventing rock burst by artificial liberation layer of roof. J. China Coal Soc..

[17] Xia Y.X., Pan J.F., Xie F., Sun X.D., Lu C., Zhang C.Y., Liu S.H. (2022). Mechanism and effect of rock burst prevention using overlength horizontal hole staged fracturing technology. J. China Coal Soc..

[18] Zhuang J., Mu Z., Cai W., He H., Hosking L.J., Xi G., Jiao B. (2024). Multistage hydraulic fracturing of a horizontal well for hard roof related coal burst control: Insights from numerical modelling to field application. Int. J. Min. Sci. Technol..

[19] Zou J., Zhang Q., Jiang Y., Jiao Y.Y., Zhu S.T., Zhang G.H. (2024). Mechanism of hydraulic fracturing for controlling strong mining-induced earthquakes induced by coal mining. Int. J. Rock Mech. Min. Sci..

[20] Tian X., Gong S., Tang C., Dou L.M., Zhang R.P. (2025). Research on the Construction of Three-Dimensional Longitudinal Wave Velocity Model Based on Underground-Surface Joint Microseismic Monitoring. Rock Mech. Rock Eng..

[21] Li Z.L., Dou L.M., Cai W., Wang G.F., Ding Y.L., Kong Y. (2016). Roadway stagger layout for effective control of goaf-side rock bursts in the longwall mining of a thick coal seam. Rock Mech. Rock Eng..

[22] Dou L.M., Cao J.R., Cao A.Y., Chai Y.J., Bai J.Z., Kan J.L. (2021). Research on types of coal mine tremor and propagation law of shock waves. Coal Sci. Technol..

[23] Jiang C.S., Zhao Y.Z., Wang X.Z. (2010). Periodic characteristics of Benioff strain release and strong earthquake activity in Asia. Earthquake.

[24] Cai W., Bai X., Si G., Cao W.Z., Gong S.Y., Dou L.M. (2020). A Monitoring Investigation into Rock Burst Mechanism Based on the Coupled Theory of Static and Dynamic Stresses. Rock Mech. Rock Eng..

[25] Benioff H. (1951). Earthquakes and rock creep:(Part I: Creep characteristics of rocks and the origin of aftershocks). Bull. Seismol. Soc. Am..

[26] Cai W., Dou L.M., Si G.Y., Cao A.Y., Gong S.Y., Wang G.F., Yuan S.S. (2019). A new seismic-based strain energy methodology for coal burst forecasting in underground coal mines. Int. J. Rock Mech. Min. Sci..

[27] Martin C.D., Chandler N.A. (1994). The progressive fracture of Lac du Bonnet granite. Int. J. Rock. Mech. Min. Sci. Geomech. Abstr..

[28] Zhao Y., Yang T., Zhang P., Zhou J., Yu Q., Deng W. (2017). The analysis of rock damage process based on the microseismic monitoring and numerical simulations. Tunn. Undergr. Space Technol..

[29] Ding W., Tan S., Zhu R., Jiang H., Zhang Q. (2021). Study on the Damage Process and Numerical Simulation of Tunnel Excavation in Water-Rich Soft Rock. Appl. Sci..

[30] Zhang E.F., Yang G.S., Tang L.Y., Yang Q., Xie Z.W. (2019). Study on influence of water content to damage and degradation laws of argillaceous siltstone. Coal Sci. Technol..

